# Chronic Inflammation—A Link between Nonalcoholic Fatty Liver Disease (NAFLD) and Dysfunctional Adipose Tissue

**DOI:** 10.3390/medicina58050641

**Published:** 2022-05-06

**Authors:** Maria Petrescu, Sonia Irina Vlaicu, Lorena Ciumărnean, Mircea Vasile Milaciu, Codruța Mărginean, Mira Florea, Ștefan Cristian Vesa, Monica Popa

**Affiliations:** 1 Department of Community Medicine, Faculty of Medicine, “Iuliu Hațieganu” University of Medicine and Pharmacy, 400349 Cluj-Napoca, Romania; maria.petrescu@umfcluj.ro (M.P.); codruta.marginean@yahoo.com (C.M.); miraflorea@yahoo.com (M.F.); dr_monica_popa@yahoo.com (M.P.); 2 5th Department–Internal Medicine, 1st Medical Clinic, Faculty of Medicine, “Iuliu Hațieganu” University of Medicine and Pharmacy, 400012 Cluj-Napoca, Romania; vlaicus@yahoo.com; 3 5th Department–Internal Medicine, 4th Medical Clinic, Faculty of Medicine, “Iuliu Hațieganu” University of Medicine and Pharmacy, 400015 Cluj-Napoca, Romania; lorena_ciumarnean@umfcluj.ro (L.C.); mircea_milaciu@yahoo.com (M.V.M.); 4 2nd Department–Functional Sciences, Discipline of Pharmacology, Toxicology and Clinical Pharmacology, Faculty of Medicine, “Iuliu Hațieganu” University of Medicine and Pharmacy, 400337 Cluj-Napoca, Romania

**Keywords:** liver, inflammation, adipocytes, molecular pathways, fibrosis

## Abstract

Nonalcoholic fatty liver disease (NAFLD) is a new challenge in modern medicine, due to its high prevalence in the world. The pathogenesis of NAFLD is a complex dysmetabolic process, following the “multiple-hit” hypothesis that involves hepatocytes excessive accumulation of triglycerides, insulin resistance (IR), increased oxidative stress, chronic low-grade inflammatory response and lipotoxicity. In this review, we provide an overview of the interrelation of these processes, the link between systemic and local inflammation and the role of dysfunctional adipose tissue (AT) in the NAFLD development. Multiple extrahepatic triggers of the pathophysiological mechanisms of NAFLD are described: nutritional deficiency or malnutrition, unhealthy food intake, the dysfunction of the liver–gut axis, the involvement of the mesenteric adipose tissue, the role of adipokines such as adiponectin, of food intake hormone, the leptin and leptin resistance (LR) and adipose tissue’s hormone, the resistin. In addition, a wide range of intrahepatic players are involved: oxidative stress, fatty acid oxidation, endoplasmic reticulum stress, mitochondrial dysfunction, resident macrophages (Kupffer cells), neutrophils, dendritic cells (DCs), B and T lymphocytes contributing to the potential evolution of NAFLD to nonalcoholic steatohepatitis (NASH). This interdependent approach to complex dysmetabolic imbalance in NAFLD, integrating relevant studies, could contribute to a better clarification of pathogenesis and consequently the development of new personalized treatments, targeting de novo lipogenesis, chronic inflammation and fibrosis. Further studies are needed to focus not only on treatment, but also on prevention strategy in NAFLD.

## 1. Introduction

NAFLD is an epidemic liver disease, affecting almost one quarter of the world’s population, and represents the second cause of liver transplantation in the USA [1,2]. Furthermore, while the prevalence of NAFLD in the normal weight patient population is between 20 and 30%, in the obese population, NAFLD occurs between 75 and 100% of subjects [3,4]. A recently published meta-analysis on obese patients in Europe found a NAFLD incidence of 57.03% and NASH incidence of 81.83% [3].

NAFLD develops due to hepatic accumulation of lipids, which in turn can progress to its severe form, NASH. It is unrelated to alcohol ingestion or hepatic viral infections [5,6,7,8]. NAFLD is characterized by an increase in hepatic triglycerides (60% from albumin-bound fatty acids, 15% from diets, 25% locally synthesized), biochemically by an increase in triglycerides (TG) level and by the presence of more than 5% hepatic lipids as detected by magnetic resonance imaging (MRI) [9,10,11]. Current data have suggested that two pathogenic pathways are involved in the development and progression of NAFLD: (1) lipotoxicity that induces mitochondrial abnormalities and will increase liver sensitivity to liver inflammatory markers; and (2) enhanced lipid peroxidation (LPO) by the reactive oxygen species (ROS) [4]. Two hypotheses have been described in NAFLD. One hypothesis is called the “two-hit model”, being made up of the “first hit” represented by liver steatosis and the “second hit”, which represents the progression of steatosis to steatohepatitis. The other hypothesis is called “multiple parallel hits” and refers to the inter-relationship of IR, lipotoxicity, oxidative stress (OS), endoplasmic reticulum (ER) stress (ERS), and gut-microbiota dysfunction [2,9].

The chronic low grade inflammation is considered to play a dominant role in the potential development of severe form, NASH and liver complications (fibrosis, cirrhosis, liver cancer) and extrahepatic complications (cardiovascular diseases, type 2 diabetes mellitus, renal dysfunction) [12,13]. In NASH, the inflammation could be promoted by IR, dyslipidemia, and peripheral AT dysfunction, leading to potential irreversible lesions [14,15]. There is a certain category of patients who do not have a progression toward cirrhosis and cancer [16].

In this review, we aim to provide an overview of the interrelation of these processes, focusing on the link between systemic and local inflammation, and the role of dysfunctional AT in the NAFLD development.

## 2. Triggers of Liver Inflammation

### 2.1. Extrahepatic Triggers of Inflammation

#### 2.1.1. Inadequate Calorie Intake and Nutrition Deficiencies

Consistent with two hypothesis, inadequate caloric intake and nutritional deficiencies lead to the development of NAFLD and its severe form, NASH. Many studies evaluated both the period of the unhealthy food behavior and food composition.

A mice study assessed the degree of histological proven steatosis and inflammation in relation to unhealthy eating and its period. A high degree of steatosis was found after 52 weeks compared to 24 weeks of inadequate food intake. Moreover, the level of inflammatory histopathological areas and hepatocyte ballooning were found higher after 40 weeks of unhealthy eating compared to 24 weeks [17]. The study of liver damage experimentally induced by methionine and choline deficient diets (MCD) was shown to peak at 8 weeks of chemokine CC motif ligand 2 (CCL2) and matrix metalloproteinase (MMP)-13. The authors concluded that MCD diets under 16 weeks were associated with a reversible NASH, which became irreversible after 16 weeks of MCD intake [18].

In terms of dietary habits, industrial trans-unsaturated diets stimulated sterol regulatory element binding protein (SREBP) pathway and activation of SREBP cleavage-activating protein (SCAP)-SREBP axis, leading to cholesterogenesis and NAFLD [19]. Another study showed that lipogenesis was reversed after SREBP-1c activity inhibition by antisense oligonucleotide (ASO), but without effects of ASO on insulin signaling [20]. Sugar ingestion leads to the activation of carbohydrate responsive element-binding protein (ChREBP). In terms of IR, the action of ChREBP is controversial. One study showed that knockout of ChREBP increased insulin sensitivity while others showed that increased ChREBP led to insulin sensitivity. Fructose stimulates steatosis dependently of ChREBP [21,22]. Hepatic ChREBP suppress the SREBP2 activity leading to regression of liver inflammation. The systemic knockout of ChREBP led to profound hepatic inflammation [23,24]. It was documented that fructose interacted with some amino-group proteins in the generation of advanced glycation-end products through nuclear factor kappa B (NF-κB) pathway inflammation [25]. Recently, studies tried to evaluate this potential therapeutical target through inhibition of NF–κB pathway and found a regression of NAFLD (Table 1) [26]. Fructose is considered a hepatotoxin, leading to OS, which has been alleviated (by NADPH oxidases [NOXs] suppression) following the experimental administration of magnesium (Table 1) [27]. Another study evaluated the relationship between ChREBP and fibroblast growth factor 21 (FGF21) following fructose ingestion and found a protective role of FGF21 regarding inflammation, but the presence of ChREBP is needed to become active. It was concluded that the axis between ChREBP and FGF21 can contribute to the pathogenesis of NAFLD [23]. FGF21 has become a therapeutic target, as shown by a study using experimental astaxanthin administration (a carotenoid) that led to improvement of mitochondrial function through regulation of peroxisome proliferator-activated receptor gamma (PPAR-γ) coactivator-1α (PGC-1α) made by FGF21 (Table 1) [28]. Diets in saturated fatty acids led to changes in the plasma of circulatory composition of FFA and contribute to the development of inflammation in NAFLD [29].

High protein and carbohydrate diets, saturated fatty acids (SFA) and monounsaturated fatty acids (MUFA) have been reported in NASH patients that had low polyunsaturated fatty acids (PUFA) and total lipids. SFA and MUFA have implications for OS. Elevation of total protein levels could be another dominant factor in the development of NASH. High levels of carbohydrates, especially fructose, worsen insulin sensitivity and increase de novo lipogenesis (DNL). Unbalanced lipid intake leads to alterations in redox status and ROS production [30,31].

An experimental murine study compared Amilin liver NASH (AMLN) diet and choline-deficient L-amino acid defined (CDAA) diet with normal diets. In terms of lipid metabolism, AMLN overdose led to mitochondrial dysfunction. Both dietary patterns led to the activation of inflammatory markers, especially interleukin (IL)-6, which appears to have been implicated in reducing insulin signaling. In terms of fibrogenesis, both diet models were involved [32]. All these elements have shown the important role of SREBP-1 and ChREBP in the development of NAFLD and NASH elements due to inappropriate eating behavior.

There are also studies with sanogenic diets and their influence in the NAFLD and NASH. One of them is diet containing polyphenols, which appears to act by increasing aerobic lipid metabolism [33], and another one is with tetrahydrocurcuminum. An experimental study showed that curcuminum impaired the phosphorylation of insulin receptor substrate 1 (IRS1)/phosphatidylinositol 3-kinase(PI3K)/protein kinase B(Akt) and also downstream signaling pathways, forkhead box protein O1 (FOXO1) and glycogen synthase kinase 3β (GSK3β) [34].

Most of the studies cited are focused on the attempt to elucidate the pathophysiological mechanisms involved in the sequence of events from simple steatosis to NASH, cirrhosis, and, eventually, to hepatocellular carcinoma (HCC) [18,35]. However, given some contradictory results, further studies are needed to elucidate them.

**Table 1:** Drugs that limit inflammation in NAFLD development and its more sever condition, NASH.

Target	Drug	Level of Confidence (In Vivo, In Vitro, Clinical Trial)	Effect	Reference
Modulation of Leptin/adiponectin axis	LMF–HSFx	In Vivo	Enhances leptin and adiponectin in adipocyte Decreases IR	[36]
Reduce ROS Anti-inflammatory	Magnesium isoglycyrrhizinate	In Vivo	Down-regulating mRNA and protein levels of NOX1, NOX2 and NOX4	[27]
Anti–inflammatory	Antrodan	In Vivo	Enhances AMPK/Sirt1/SREBP-1c/PPARγ pathway activity	[37]
Anti-inflammatory Increase hepatic lipolysis Prevention of lipogenesis	Ginsenosides	In Vivo	Gut leakage endotoxinemia	[38,39]
Anti–inflammatory Reduce OS	Diceratella elliptica	In Vivo	Nrf2 inducer	[40]
Reduce OS	Flinax	In Vivo	Restores the efficiency of mitochondrial function	[41]
Anti-inflammatory Anti-apoptosis Prevention of fibrosis	Scoparone	In Vivo	Blocking TLR4/NF–κB pathway Blocking ROS/P38/Nrf2 axis Blocking PI3K/AKT/mTOR pathway	[42,43]
Anti-inflammatory, Reduce OS	Ruzu herbal bitters	In Vivo	Inhibit of NF–κB pathway	[26]
Anti-inflammatory Reduce OS Anti-tumor	Astaxanthin	In Vivo In Vitro	Up-regulate FGF21/PGC-1α pathway	[28]
Anti-inflammatory in KCs and hepatocytes	Naringerin	In Vivo	Down–regulating NLRP3/NF–κB pathway	[44]
Anti-inflammatory Reduce OS Reduce lipid depots	Polydatin	In Vivo	Inhibit Keap1 Increase Nrf2 activity	[45]

#### 2.1.2. Metabolic Dysfunction

IR leads to liver OS, disturbance of lipoprotein lipase (LPL) and mitochondrial oxidation of free fatty acids (FFA) [46]. It is well known that IR is strongly associated with NAFLD, but it is not clear if IR is a cause or a consequence of NAFLD. IR could be a trigger of inflammation in NAFLD, as shown on an experimental study in which inflammation was induced by iron therapy. They have concluded that there is a direct proportional relationship between IR and liver inflammatory markers and an inverse relationship between IR and high density lipoprotein (HDL) cholesterol [47,48,49,50]. AT distribution according to gender plays an important role in the development of NAFLD because of various mediators, named adipokines. Adiponectin, one of the most important players in NAFLD, is mainly produced by adipocytes. Adiponectin correlated negatively with IR. Furthermore, adiponectin has a protective role in inflammation and antisteatogenic role through 5–AMP kinase, leading to inhibition of acetyl-CoA decarboxylase (ACC) and fatty synthase. It also decreases the influx of fatty acids to liver (Table 2) [51]. Modulation of adiponectin was performed by experimental administration of low molecular weight fucoidan and high stability fucoxanthin (LMF-HSFx) (Table 1) [36]. Antrodan is another experimental drug with a role in increasing adiponectin levels and thus improving NAFLD (Table 1) [37].

**Figure 1:**
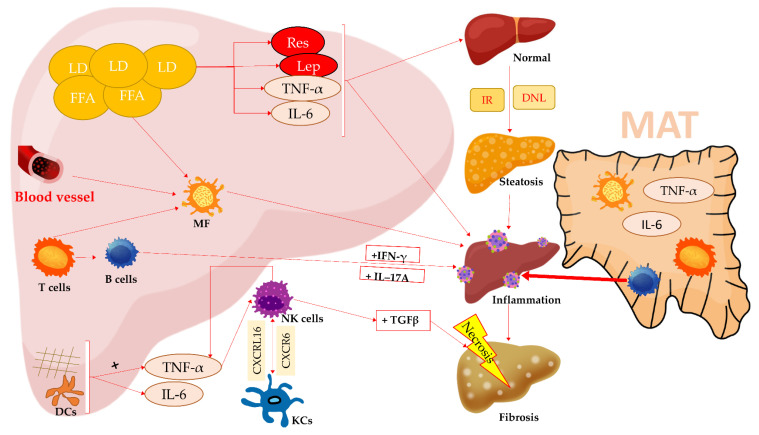
The molecular players during progression from NAFLD and its severe form, NASH.

Leptin is a peptide hormone secreted mainly by white adipose tissue (WAT) involved in reducing food intake in. In obesity, the body develops LR, probably because of a mutation of leptin receptors. Regarding the relationship between leptin and its receptor, authors evidenced in a human observational study the existence of a negative relationship—probably because of a negative feedback, or possibly because of a peripheral resistance in leptin action [52]. This idea was reinforced by others who postulated that hyper-leptinemia led to peripheral or central LR and promotes steatosis and IR of prediabetes patients with or without NAFLD [53]. Experimental administration of ginsenside (the root and rhizome of *Panax ginseng*) led to LR improvement. Along with improving LR, ginsenoside has also been shown to have beneficial effects on liver inflammation and lipolytic genes (Table 1) [38,39]. Higher values of postprandial leptin were also associated with the degree of liver steatosis, considering a close relationship between IR and pro-inflammatory cytokines. Regarding insulin signaling, an original observational study found an association between serum leptin levels and NAFLD of prediabetic patients. They considered that this association is the consequence of insulin secretory dysfunction and IR of prediabetic patients (Table 2). Concerning the relationship between leptin and inflammatory component of NAFLD, another study found that leptin increases the pro-inflammatory and pro-fibrotic effects. As new therapeutical strategies, another experimental in vivo study administered LMW-HSFx and found a decrease of IR through higher leptin expression in adipocytes, and, consequently, decreased IR (Table 1) [36,54,55]. There are some studies that found a significant relationship between white blood cell (WBC) number, IR and steatosis. FFA leads to lipid peroxidation and cytokine production, which leads to high values of WBC [56]. In addition to modulating leptin activity in the liver, it also acts in the intestine by decreasing tight junction proteins in intestinal epithelial cells in vivo and in vitro (Figure 1) [57].

Resistin, another adipokine, has an important role in steatosis, IR and inflammation. In terms of inflammation, resistin activated the NF-κB pathway and led to the increase in pro-inflammatory cytokines (Table 2). In high-fat diet (HFD) animal model, resistin led to abnormal mitochondrial activity and up-regulated pro-inflammatory factors. All these happened through AMP activated kinase (AMPK)/PGC-1α pathway [58].

Visfatin, a protein that is preferentially produced in visceral AT contributes to IR (reduce the proteins involved in insulin signaling) and inflammation through Janus kinase 2 (JAK2)/signal transductor and activator of transcription 3 (STAT3) and IκB kinase (IKK)/NF-κB pathway. Experimental inhibition of JAK2 and NF-κB led to lowers mRNA levels of IL-6 and tumor necrosis factor (TNF)-α and also rescued insulin signaling [59]. Hepatic lipid infiltration and pro-inflammatory effects were also found to be given by visfatin. The pro-inflammatory effect was associated with the fact that visfatin increased serum leukocytes levels [60]. While the last two studies cited found a directly proportional relationship between visfatin and inflammation, the next study showed an inversely proportional relationship between visfatin and TNF-α [61].

Hepatic lipids triggers inflammation through TNF-α and IL-6 activation. Liver lipid droplet accumulation and FFA liver influx lead to steatosis and MF activation. T cells contribute to liver inflammation through pro-inflammatory cytokines such as IFN-γ and IL-17A. Likewise, activation of CD68^+^ MF and B cells promote liver inflammation, injury and fibrosis. The CXCR6 and its cognate CXCL16 are expressed by hepatocytes and promote the interaction between NK cells and KCs and augment inflammation. Fibrotic cells together with DCs induce TNF-α activation and IL-6 release. DCs, together with TNF-α, cause NK cells activation which potentiates further TNF-α activity. NK cells interact with TGF-β, contributing to cellular death. MAT contains inflammatory markers such as TNF-α, IL-6 and a multitude of immune cells: T lymphocytes, B lymphocytes and MF. B lymphocytes are able to migrate into the liver and induce inflammation. Abbreviation: Res, resistin; Lep, leptin; MAT, mesenteric adipose tissue; MF, macrophages; LD, lipid droplet; FFA, free fatty acids; T cells, T lymphocytes; B cells, B lymphocytes; DCs, dendritic cells; TNF-α, tumor necrosis factor α; IL-6, interleukin 6; IL-17A, interleukin 17A; CXCR16, C-X-C motif receptor 16; CXCL16, C-X-C motif chemokine 16; KCs, Kupffer cells, NKc, natural killer cells; IFN-γ, interferon-γ; TGFβ, transforming growth factor-β; IR, insulin resistance; and DNL, de novo lipogenesis. Elements used and edited with permission of iStocks.

Adipsin (complement factor D) is a serine protease synthesized by adipocytes, contributes to the alternative pathway of complement activation. It is one of the main proteins of the adipose cells, that catalyzes the rate limiting step of alternative pathway of complement activation [62]. It is highly and almost exclusively expressed in AT and could lead to remission of NAFLD lesions. It was found a positive correlation with homeostasis model assessment method for IR (HOMA-IR) and also with insulin levels. An experimental study found that adipsin ablation led to an increase of AT inflammation [62,63]. Another cohort human study included 908 patients with NAFD detected by abdominal ultrasonography and followed for three years. It found that adipsin levels were inversely associated with NAFD remission and postulated that adipsin could be a novel biomarker in NAFLD remission [63]. Less is known about its liver inflammatory action. One human study found that complement factor D was associated with high levels of C–reactive protein (CRP) on patients with chronic heart failure [64].

Omentin is mainly expressed in the vascular fraction of visceral fat depot [65]. The adipokine facilitates insulin-stimulated glucose transport through activating PI3K-AKT pathway, which plays a main role in cell metabolism, growth and survival [66]. In a human study of NAFLD evidenced by biopsy, authors found that omentin was associated with the hepatocyte ballooning degeneration. Regarding IR a negative correlation was found, but there was a positive correlation with inflammatory markers. Further studies are needed to establish the relationship between high sensitivity (hs)-CRP and omentin [65]. Another study found a positive relationship between omentin levels and the liver degree inflammation in NAFLD based on liver biopsy [67].

Vaspin, a member of serine protease inhibitor family is an insulin-sensitizing adipokine which upregulated in metabolic disorders to compensate insulin signaling and also in inflammation. Authors suspected that vaspin might have antagonized the effects of proteases that were up-regulated in IR. In line with that, they performed a human observational study and confirmed their hypothesis, finding a positive correlation between vaspin and inflammatory markers and IR (Table 2) [68]. Regarding fibrosis, a feature of NASH, Aktas et al. found a positive correlation between vaspin and the degree of liver fibrosis. Evidence regarding the relationship of vaspin with inflammatory markers appears inconclusive at the moment, with positive correlations, or no association found [67,69]. Further studies are likely to be needed to determine the exact reaction between adipokines and inflammation.

**Table 2:** The relationship between adipokines and liver or adipose tissue inflammation.

Adipokines	Effect on Liver or AT Inflammation	Mechanism	References
Adiponectin	Liver anti-inflammatory	Downregulate TNF–α Inhibit MF Inhibit T cells function and proliferation Inhibit IL–1 and IL-6 signaling	[51]
Leptin	Liver pro–inflammatory	Upregulate MF, neutrophils, T cell, NK cell function Upregulate the TNF–α and IL-6	[51,55]
Resistin	Liver pro–inflammatory	Increase mRNA expression of IL–6, IL-1β, TNF–α, CRP, and soluble adhesion molecules Increase infiltration of MF to liver tissue Activates NF–κB factor	[58]
Visfatin	Liver pro–inflammatory Liver anti-inflammatory	High levels of IL–6, TNF–α, IL-1β Increase expression of MMP–9, IL–8, TNF–α Induce MMP–9 in MF Negatively correlation with TNF-α	[59,61,70]
Adipsin	AT pro–inflammatory	High MF genes (*Cd11b, Cd11c, F4/80, Mac2*) High mast cell genes (*Mcpt4, cma1, cpa3*)	[62,64]
Vaspin	Liver pro–inflammatory	High values of hs–CPR and IL-6	[68,69]

Abbreviation: CRP, c–reactive protein; MF–macrophages; hs, high sensitivity.

### 2.2. Intrahepatic Triggers of Inflammation

#### 2.2.1. Oxidative Cellular Damage

In a comparative study authors evaluated the OS induced cellular damage in NAFLD, consistent to multiple parallel hits hypothesis [71]. They compared hepatic levels of 4-hydroxy-2-noneal (HNE) and 8-hydroxydeoxyguanosine (8-OHdG), markers for LPO and oxidative DNA damage, respectively, between 23 NAFLD patients, 17 NASH patients, and also 7 subjects with normal liver. 8-OHdG expression was detected in 2 NAFLD subjects and in 11 of the NASH patients, while HNE expression was detected in 18 NAFLD patients and in all 17 patients with NASH. They concluded that LPO and oxidative DNA damage frequently occurred in NAFLD [71]. Another experimental study found that obese and hyperphagic rat model showed increased hepatic OS compared with non-hyperphagic rat model. Additionally, chemokine systems are also related to liver OS [11,72]. NLR family pyrin domain-containing 3 (NLRP3) activity occurs in concert with other pathways, including oxidative stress. NLRP3 once inhibited experimentally led to reversal of NSAH lesions [73]. Chemokines are ubiquitous chemotactic molecules with a dominant role in acute and chronic inflammation. C-X-C motif receptor 3 (CXCR3) is highly expressed in T lymphocytes, monocytes, and macrophages (MF), etc. It serves as a receptor for C-X-C motif chemokines (CXCL). One of the chemokines is CXCL9 which interacts with monokine induced by interferon-gamma (MIG) to modulate the CD4^+^CD24^+^ regulatory T (Treg) cells/Th17 ratio. The experimental inhibition of the interrelationship between MIG and CXCL9 led to increased Treg/Th17 ratio and improved OS [74]. Another chemokine that binds to CXCR3 is CXCL10. It is a strong ligand that binds lipotoxic hepatocytes to MF and, ultimately, activates the inflammatory cascade [75]. In addition to activating the inflammatory cascade, another study showed that CXCL10 is also involved in modulating the autophagic pathway in NAFLD [76]. Last but not least, CXCL11 resulted in elevated peripheral blood mononuclear cells (PBMCs) and was positively correlated with CXCR3 levels, authors concluding a response of liver cells to high CXCL11 levels [77]. Another chemokine involved in the evolution of NAFLD to fibrosis is CXCR6 along with its ligand, CXCL16, that accumulates in natural killer (NK) cells. Inflammatory dominance of MF is considered by some authors as a response to the interaction between CXCR6-NK cell accumulation in the liver (Figure 1) [78]. Several animal studies found the positive correlation between CXCR3 expression and 8-OHdG levels in the liver [11,72]. Other studies tried to postulate that the length of leukocyte telomere could be a predictor of NAFLD, as it is known that leukocytes are involved in OS [79,80]. A consequence of OS in NAFLD is the release of epitopes that play a role in modulating the immune response and generating pro-inflammatory cytokine [1]. In an attempt to develop new therapeutic means in NAFLD, authors evaluated the beneficial effects of scoparone and found its action on Toll like receptors (TLR)-4/NF-κB axis with the improvement of pathophysiological elements, including OS (Table 1) [43]. Other studies have shown no improvement in OS after experimental administration of naringerin, which is known to act on the NLRP3/NF-κB axis, but has found an improvement of inflammation in Kupffer cells (KCs) and hepatocytes (Table 1) [44]. OS modulation continues to be of interest. Some authors focusing that the experimental administration of *Diceratella elliptica* stimulated nuclear factor erythroid 2-related factor 2 (Nrf) activity, reduced OS, and, in the end, phosphorylation of insulin receptors [40]. Another way to modulate OS was Kelch-like ECH-associated protein 1 (Keap1)/Nrf2 pathway. Fructose increased Keap1 expression and Nrf2 blockade and OS, while experimental administration of polydatin reversed these effects (Table 1) [45].

#### 2.2.2. Fatty Acid Oxidation (FAO)

Lipid catabolism in the liver is strongly dependent on mitochondrial metabolism, through FAO. The mitochondrial metabolism disorders and decrease of FAO leads to FFA accumulation in the liver and NAFLD occurrence. This was supported by the animal study that demonstrate Methylation-Controlled J (MCJ) protein, a transmembrane protein in the inner mitochondrial membrane, acts as an endogenous negative regulator of the respiratory chain Complex I [81]. Synthetic small interference RNA (siRNA)-mediated depletion of MCJ reduces hepatic steatosis and fibrosis in multiple NASH mouse models associated with increased β-oxidation [82]. Another beneficial player in FAO modulation is represented by farnesol, which undergoes dephosphorylation and leads to non-sterol isoprenoid farnesol (FOH). Farnesol has been considered a key element in cholesterol biosynthetic pathways. In addition to the aforementioned FAO modulation, it also reduces the expression of fatty acid synthesis genes, through farnesoid-x-receptor (FXR) and PPAR. It could be considered a possible therapeutic target for NAFLD [83]. Another study showed that caspase 1 and neutrophil elastase inhibition played beneficial roles [84]. Modulation of PPARα activity is an important player in FAO. One of the PPARα modulators is PI3K (more exactly, the PIK3K subunit) and found regression of NAFLD lesions, while its inhibition in normal liver mice led to NAFLD [85]. Another element in FAO modulation is represented by poly (ADP ribose) polymerase 1 (PARP1), also having as substrate PPARα. It inhibits PPARα activity and led to reduction of FAO. Experimental reduction in PARP1 activity found an improvement in PPARα activity, increased FAO, limited steatosis and inflammation [86]. Last but not least, zinc finger protein 300 (ZNF300) intervenes in the modulation of PPARα activity by KRAB domain encoding and 12 C2H2 type zinc finger domain. It could induce PPARα expression through directly binding to its promoter [87]. An important role in FAO modulation is played by β-oxidation-SMAD signaling, which regulates the expression of β genes, being related to the induction of lipid accumulation by palmitate (Figure 1) [88].

#### 2.2.3. Endoplasmic Reticulum Stress (ERS)

The ER is a multifunctional membrane with an important role in protein maturation. Lipid accumulation leads to misfolded protein accumulation in ER lumen and ERS. The ER tries to respond to stress through PERK–eIF1α–ATF4, IRE1–XBP1 or ATF6 molecular branches. Without the possibility of ER homeostasis restoration, NAFLD occurs. A bridge between ERS and steatosis is represented by Forkhead box A3 (FOXA3), a member of FOXA family. An experimental study showed that only the transcriptional factor XBP1c under ERS interfered with the modulation of FOXA3 activity and led to lipid synthesis while FOXA3 inhibition reversed the lesions and improved ER activity even under HFD diets. No changes were found regarding the link between FOXA and inflammation, but experimental inhibition of FOXA3 reduced the inflammation [89]. Another element associated with ERS is autophagy, with its subtype, lipophagy, with a well-established role in energy homeostasis and hepatocyte lipid content, leading to NAFLD. Experimental administration of high carbohydrate diets (HCD) to yellow fish led to increased Ca^2+^ fluxes and up-regulated the ERS markers. This was reversed by the administration of 4-Phenylbutiryc acid (4-PBA). It also reduced the mRNA expression of lipogenic genes and increased lipolytic genes [90]. The relationship between ERS and NAFLD pathogenesis is demonstrated by beneficial effects of *Zingiber afficinale* extract along with omega-3 fatty acid in an experimental study in Wistar rats that followed 8 weeks of HFD, subsequently recived zingiber. A reduction in the expression of lipogenic and ERS genes was found [8]. Strontium is another ERS modulator in NAFLD. Using an in vitro NAFLD model with human hepatocyte cell line (L02) treated with palmitic acid (PA), it was found that experimental administration of strontium decreased lipogenic genes, TG synthesis with the improvement of ERS and steatosis [91]. Some of the modulating elements of ERS stated in our paper could serve as possible therapeutic targets in preventing NAFLD and its severe form, NASH [8,90,91].

#### 2.2.4. Mitochondrial Dysfunction

Mitochondria is the main energy source of hepatocytes and play a major role in extensive oxidative metabolism and normal function of the liver [92]. Mitochondria contain a double membrane structure [93]. Regarding the mitochondrial inner membrane, an article evaluated the relationship between Ant2 and mitochondrial respiration and found that experimental inhibition of Ant2 increased mitochondrial mass, improved steatosis and IR but without any effects on inflammation [94]. Experimental growth of the lipid droplet coat protein Perilipin 5 (PLIN5) expression in brown adipose tissue (BAT) improved insulin sensitivity, inflammatory activity and mitochondrial function [95]. The mitochondrial outer membrane contains certain proteins, including Sam50 which is modulated by sorting and assembly machinery (SAM) complex protein (SAMM50) with a role in regulating oxidative activity, mitochondrial morphology and mitophagy. An observational study found that decreased SAMM50 in encoding Sam50 led to decreased FAO and increased lipid accumulation. The effects were reversed after increasing SAMM50 levels [96]. Some study evaluated the involvement of mitochondrial membrane potential (ΔΨm) in hyperglycemic status on HepaG2 cells. Hyperglycemia led to hyperpolarization of the membrane, mitochondrial dysfunction and high risk of the collapse of potential membrane with the onset of apoptosis. These have been reversed after experimental administration of phenols. Further studies are needed to explore the mechanism by which constituents of mulberry with a protective role in mitochondrial function of hyperglycemic liver cells [97]. Another savior of mitochondrial function is represented by a mixture of nutraceuticals (i.e., vitamin E, vitamin D3, olive-dry extract, cinnamon dry-extract and fish oil). They led to increase in respiratory chain activity and mitochondrial ATP production, and, finally, the improvement of NAFLD [41]. Another in vitro model study evaluated the role of polyphenols on modulation of mitochondrial activity and found an improvement of mitochondrial function through sirtuin 1 (SIRT1) activity and the deacetylation of PGC-1α [33]. The perturbation of the FGF21/PGC-1α axis contributes to mitochondrial dysfunction. Experimental administration of Astaxanthin, increased this axis with improved mitochondrial function [28]. With respect to temporal relationship between IR and mitochondrial dysfunction, it was found that mitochondrial dysfunction precedes IR and NAFLD development in an experimental rats study [98].

Additionally, related to mitochondrial function, another element in its modulation is CXCR3. The experimental CXCR3 inhibition restored mitochondrial function and inhibited mitochondrial-dependent apoptosis in mice liver fed MCD diet and hepatocytes cell line [72]. Another positive element in restoring the function and number of mitochondria is the experimental depletion of neutrophil activity [99].

## 3. AT Dysfunction in NAFLD

The role of AT in NAFLD is not fully understood. Inflammation of specific depots of WAT has a key role in NAFLD progression [100]. In terms of markers, the literature mentions that the ratio of leptin to adiponectin is a suggestive of visceral adiposity [101]. There are several places of fat distribution: mesenteric adipose tissue (MAT), peritoneal adipose tissue (PAT), retroperitoneal adipose tissue (RAT), epididymal adipose tissue (EAT) and umbilical adipose tissue (OAT) [57]. Visceral WAT (vWAT) has a dual activity. As its size increased, the level of pro-inflammatory cytokines also increased and adiponectin level decreased while the fibrosis of vWAT led to decreased in adiponectin levels [102]. Subcutaneous adipose tissue (SAT) is not associated with NAFLD [29].

### 3.1. Mesenteric Adipose Tissue (MAT)

In an attempt to detect the location of dysfunctional AT, one study analyzed all sites and concluded that the MAT was rather involved in the development of NAFLD (Figure 1) [57]. Another study found that MAT is a vector for innate immunity with higher levels of T cells and MF. In the inflammatory stage of MAT, B cells migrated to the liver and increased mRNA expression of pro-inflammatory cytokines (Figure 1) [103]. In addition to modulating hepatic mitochondrial dysfunction, SIRT1 is also involved in modulating lipogenic genes in AT. Experimental SIRT1 ablation increased lipogenic genes in MAT [104], while SIRT1 experimental stimulation decreased lipogenesis. As a result, it is considered that SIRT1 could be another possible therapeutic target [105]. An experimental study postulated the idea that MAT could have a protective role in the relationship between gut and liver considering that MAT inflammation may be a compensatory response to protect the liver by maintaining intestinal integrity. On a mice model, MAT was removed and found that steatosis and inflammation were higher. Regarding intestinal barrier, they found lower levels of Zonula Ocludens–1 with higher permeability under AT stimulation and inflammation [57]. A comparison was performed between the contribution of eWAT, mWAT, inguinal or subcutaneous WAT in NASH development. After experimental mice HFD they found higher expression of eWAT. After surgical removal of eWAT, they fed another 12 weeks of HFD and found lower levels pro-inflammatory markers, but no changes in steatosis [100].

### 3.2. Other Locations of AT

As previously announced, there is no directly proportional relationship between SAT and NAFLD [102]. However, when SAT fibrogenesis occurs, it has been found to be directly proportional to the degree of obesity and NAFLD. The same proportional relationship was found regarding IR [106]. Inguinal WAT (iWAT) decreased FFA as a result of iWAT bandage storage and led to improvement of steatosis and inflammation [107].

EAT led to alteration of WAT with capsase 3 activation, followed by disturbance of AT homeostasis and its atrophy [107]. In terms of gonadal WAT, an experimental mice study with MCD diets, authors found a decrease in gonadal AT levels, suggesting that lipolysis in gonadal WAT may be related to steatosis induction [108,109]. Another experimental mice study that assessed the closed relationship between epididymal (eWAT) expansion and NAFLD found the same as the precedent that inadequate diets led to disruption of the storage capacity of eWAT. In addition, RI and glucose intolerance have been reported [110]. Regarding inflammation, Mulder et al. found that after eWAT adipocytes reached a certain size, they acquired pro-inflammatory properties leading to the development of NASH [100]. Another experimental study on retroperitoneal WAT (rWAT) found that HFD led to BMI increased followed by increased of pro-inflammatory mediators released from rWAT [111].

## 4. Conclusions

This work provides an overview of the interrelation of the complex dysmetabolic processes in the NAFLD pathogenesis. Both extrahepatic triggers (inadequate caloric and nutrition intake, metabolic dysfunction with IR, adipokines, leptin and leptin resistance, chemokines production), and intrahepatic players (oxidative stress, fatty acid oxidation, endoplasmic reticulum stress, mitochondrial dysfunction, resident macrophages) are involved in the development of NAFLD and NASH. Another major role is played by dysfunctional AT, which, depending on its distribution in the body (subcutaneous or visceral) can lead to progression of NAFLD. This interdependent approach to the complex dysmetabolic imbalance in NAFLD integrating relevant studies could contribute to a better clarification of its pathogenesis and consequently, the development of new personalized treatments, targeting *de novo* lipogenesis, chronic inflammation and fibrosis. Further studies are needed to focus not only on treatment but also on prevention strategy in NAFLD.

## Data Availability

The study did not report any novel data.
